# Hemozoin-induced immunomodulation and prostate carcinogenesis: mechanisms and implications

**DOI:** 10.3389/fonc.2026.1786079

**Published:** 2026-05-04

**Authors:** Abimbola D. Akinyosoye, Solomon O. Rotimi, Olutola E. Olasehinde, Gbolahan O. Oduselu, Solomon U. Oranusi, Olayemi O. Akinnola

**Affiliations:** 1 Department of Biological Sciences, Covenant University, Ota, Ogun, Nigeria; 2 Covenant Applied Informatics and Communication Africa Centre of Excellence (CApIC-ACE), Ota, Ogun, Nigeria; 3 Department of Biochemistry, Covenant University, Ota, Ogun, Nigeria; 4 West African Centre for Cell Biology of Infectious Pathogens (WACCBIP), University of Ghana, Accra, Ghana

**Keywords:** cancer biomarkers, chronic inflammation, hemozoin, immunomodulation, malaria, oxidative stress, prostate cancer

## Abstract

Hemozoin (Hz) is a crystalline by-product, which is produced when the Plasmodium species destroy haemoglobin, and it is commonly recognised that it leads to how our bodies respond to malaria. Although we understand pretty well its immunological actions in infectious diseases, recent research indicates that chronic exposure to Hz may actually cause cancer by sustaining inflammation and altering the immune system. In this review, we examines how the relationship between the hemozoin induced immune changes contribute to prostate cancer development arises. The presence of Hz within the macrophages and dendritic cells can modulate inflammatory signalling pathways, including NF-kB, MAPK, and STAT3, particularly in the context of co-stimulation with parasite or host-derived ligands, thereby sustaining low-grade inflammation over time. These processes can result in amplified cell growth, inhibition of cell death, and genomic instability, which are typical of cancer. In addition, while several in vitro studies have reported that the purified hemozoin elicits weak or no cytokines, it has accumulated Hz amplify responses to infections or Toll-like receptor ligands, consistent with a primarily agonistic role. Hz is seen to direct macrophages to a tumour-promoting M2, suppress cytotoxic T-cells, and increase the performance of regulatory T cells, all of which are detrimental to the immune response against tumours. The connections between Toll-like receptor (TLR) signalling, oxidative stress, and PI3K/Akt/mTOR are also investigated in relation to the possibility of encouraging tumour development in prostate conditions. Epidemiologically, malaria endemic regions also report an increase in prostate cancer incidence, though this ecological overlap is subject to major cofounding, it raises the hypothesis that repeated exposure to malaria and subsequently Hz might contribute to prostate cancer, which necessitates further studies. In addition, improved malaria control, demographic ageing, coinfections, and other factors are likely to obscure any direct association. A better comprehension of these relationships may present the possibility of novel diagnostic measures and specific interventions.

## 1 Introduction

**Figure 1:**
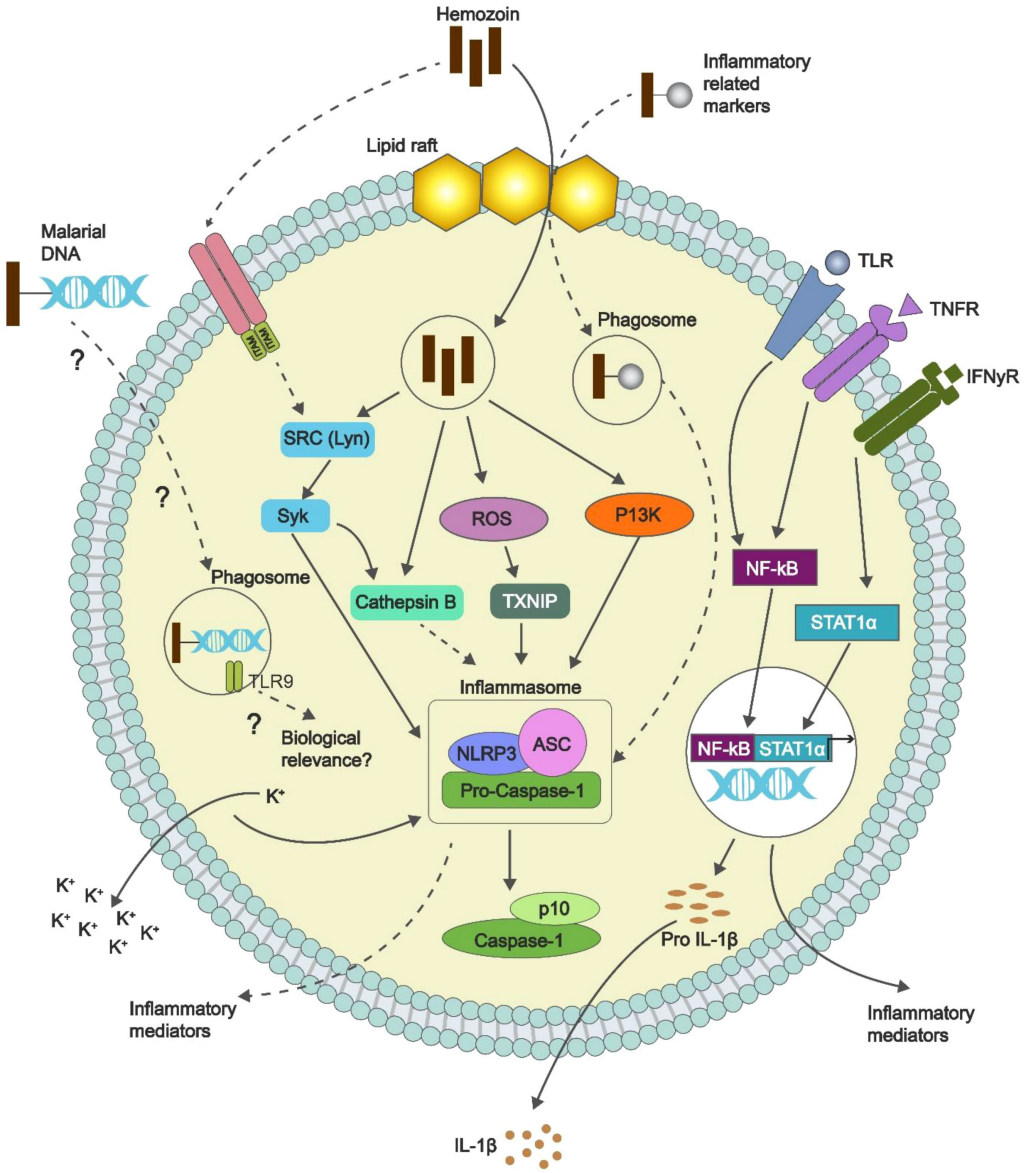
Mechanisms of hemozoin-induced immunomodulation. Hz activates the NLRP3 inflammasome, impedes dendritic cell function, and promotes a state of heightened inflammation. Adapted from (10).

Prostate cancer (PCa) is among the most commonly diagnosed cancers in men globally, with increasing rates of prevalence and mortality, especially in sub-Saharan Africa (1, 2). Although genetics, hormones and environmental factors are significant to PCa, there is an increasing focus on potential infectious agents and their by-products that may also cause the development of prostate cancer (3). One of these by-products is hemozoin (Hz) or malaria pigment, which originates in the life cycle of Plasmodium parasites when they consume haemoglobin (4, 5). Hz crystal can also alter the host’s immune response, and the immune-modulating effects of Hz now have a better understanding that includes promoting the tumour microenvironment. Recent studies suggest that Hz can activate the NLRP3 inflammasome, which causes the production and release of several pro-inflammatory cytokines, and in particular, IL-1β, TNF-α (**Figure 1**), that could contribute to immune response or tumour microenvironment (6, 7). Moreover, Hz can impede dendritic cell maturation and function, thus impeding their ability to present antigens and activate T-cells. This perpetuated immune activation and inflammation, turned on by Hz, may engender a microenvironment conducive to oncogenesis (8, 9). This situation could lead to both the start and growth of cancerous lesions in the prostate. Given that malaria is highly prevalent in areas where prostate cancer rates are increasing, it’s essential to thoroughly explore how Hz might influence the development of prostate cancer. This review seeks to bring together the latest insights on the immunological effects of Hz and its possible role in the onset of prostate cancer.

## 2 Methods

This review article is a narrative and hypothesis generating one, hence three databases (Pubmed, Scopus and Web of Science) for English articles published between 2000 to 2025 using search keywords such as hemozoin, immunomodulation, inflammasome, TLR9, Chronic inflammation, prostate cancer, tumour microenvironment, PI3K/Akt/mTOR”, “STAT3”. These results of the search was screened for key experimental, observational and reviews on hemozoin, carcinogenesis driven by inflammation and prostate cancer immunology. Where direct data on hemozoin and prostate cancer was not available, data from mechanistic studies in other organs and established models of infection associated cancer like gastric and bladder cancer were extrapolated. Due to the heterogenous nature of the study which include experimental studies, a meta-analysis was not feasible rather mechanistic themes was synthesized.

## 3 Hemozoin: biogenesis and immunological role

Hemozoin crystals are a by-product of their formation, generated during the development of Plasmodium species, especially *P. falciparum*, to soothe the toxic effects of free heme (4, 11). As the parasite catalyses the breakdown of haemoglobin in its food vacuole, a large amount of heme, an iron-containing porphyrin, is released, becoming very toxic through hydroxyl free radical or reactive oxygen species (ROS) formation and lipid peroxidation (12, 13). To protect itself, Plasmodium converts heme into an inert, insoluble crystalline form known as hemozoin through a biomineralisation process involving lipid droplets and heme–heme interactions (14).

**Table 1** summarises the crystallographic information of Hz, which is composed of iron (III)-protoporphyrin IX dimers in a triclinic crystalline structure (5). The process of dimerisation involves reciprocal iron–carboxylate bonds, which yield a stable hydrophobic aggregate resistant to enzymatic degradation by the phagocytes of the host (15, 16). They show birefringence conversions under polarised light microscopy and are chemically similar to synthetic β-hematin. Its resistance permits its persistence in tissues even after the parasitaemia has been cleared. These features highlight a biphasic interaction between Hz and the immune system. In the early stages of malaria, Hz is released at the schizont rupture stage and may transiently engage innate receptors and inflammasomes. However, this review’s hypothesis focuses on a later phase, Hz-laden phagocytes, which may persist for months or years in the endothelial reticulum and potentially in the trafficking immune cells, which therefore leads to a sustained low-grade inflammation that can influence distant organs like the prostate.

**Table 1:** Structural and physicochemical properties of hemozoin.

Property	Description
Composition	Iron (III)-protoporphyrin IX dimers
Crystal structure	Triclinic, repeating lattice
Solubility	Insoluble in aqueous and organic solvents
Detection methods	Polarised light microscopy, FTIR, Raman spectroscopy
Biological relevance	Detoxification of heme; immune system activation

**Figure 2:**
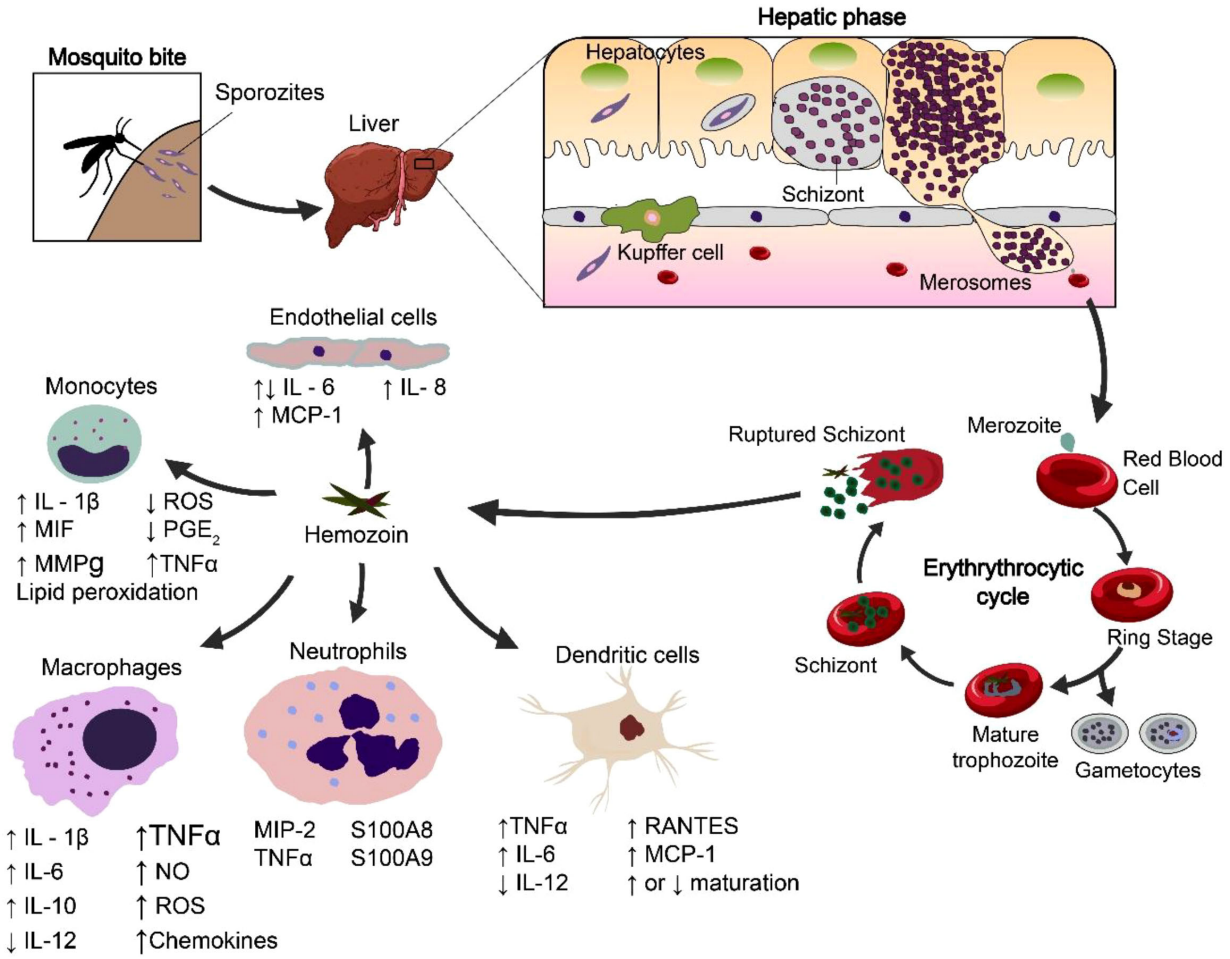
Hemozoin biogenesis and immunological activation pathways. Adapted from (19).

When infected red blood cells burst, they release hemozoin (Hz) into the bloodstream. Monocytes, macrophages, and dendritic cells phagocytose it. This phagocytosis kicks off several built-in immune responses (17, 18). Hemozoin acts as a danger signal, also known as a danger-associated molecular pattern (DAMP). It triggers the release of inflammatory proteins (**Figure 2**), including tumour necrosis factor-alpha (TNF-α), interleukin-6 (IL-6), and interferon-gamma (IFN-γ) (19, 20). These proteins play a key role in starting immune responses. However, the immunostimulatory role of Hz remains controversial. Some reports particularly highly purified B-hematin, suggest that Hz by itself is largely inert or induces only modest cytokine release and that its apparent activity may depend on contaminating parasite DNA or other ligands. In these models, Hz functions as a carrier that presents Plasmodium DNA to TLR9 or modulates responses to a second stimulus, rather than acting as a potent primary agonist. If the body makes them for too long, they can damage tissues and compromise immune function. Researchers have found that hemozoin interacts with Toll-like receptors (TLRs), TLR9. TLR9 spots unmethylated CpG DNA sequences, but experts still debate its exact mechanism (21, 22). Importantly, studies have indicated that TLR9 activation is more prominent in Hz complexed with parasite DNA. This suggests that Hz primarily allows access and presentation of DNA to endosomal TLR9, rather than activating TLR9 on its own. In this review, we therefore distinguish between the weak, acute responses to Hz and its overall immunomodulatory effect when it accumulates in the phagocytes after parasite clearance in the tissue.

Although the primary immune response of the body to hemozoin (Hz) is beneficial in overcoming parasitic infection, the consequences of constant exposure or repeated exposure may be negative (23, 24). An accumulation of Hz within the phagocytes may lead to immunological fatigue, characterised by a reduction in phagocytic activity, problems in antigen presentation, and the inhibition of co-stimulatory molecules such as CD80 and CD86 (25, 26). This immunosuppression could make the body less effective in reacting to cancer manoeuvres, even in parts of the body that are well distant from the initial malaria infection, like the prostate. Also, M2-like phenotype is adopted by macrophages loaded with Hz, which is more concerned with wound healing and immune system suppression, rather than microbial infection. This transition is crucial because M2 macrophages are thought to help tumours grow through various mechanisms like angiogenesis, remodelling of the extracellular matrix, and evading the immune response. This suggests that hemozoin might indirectly encourage cancer growth. Interestingly, hemozoin shares certain immunostimulatory properties with other crystalline materials, be it naturally occurring or externally added, such as uric acid (the monosodium urate present in gout), asbestos and silica, which can induce the inflammasome and are linked to chronic inflammation and cancer, as it is presented in **Table 2** (27).

**Table 2:** Comparative immunological effects of crystalline particles.

Particle	Source	Immune activation	Associated diseases
Hemozoin	*Plasmodium* spp.	TLRs (esp. TLR9), cytokines, ROS	Malaria, a potential carcinogenesis
Monosodium urate	Uric acid (endogenous)	NLRP3 inflammasome, IL-1β	Gout, renal disease
Asbestos	Mineral fibre (exogenous)	Macrophage activation, ROS, IL-1β	Mesothelioma, lung cancer
Silica	Industrial exposure	TNF-α, IL-6, NLRP3 inflammasome	Silicosis, lung cancer

Hemozoin isn’t just a pigment linked to malaria; it actually plays a significant role in how our immune system operates. When it lingers in phagocytes, it can lead to chronic inflammation, spur the release of cytokines, and cause immune polarisation, factors that are well-known to initiate and promote cancer development (10, 28). Understanding how hemozoin is produced and its impact on the immune response is crucial for grasping its potential involvement in diseases beyond malaria, such as prostate cancer.

## 4 Chronic inflammation and cancer development

Within the Hz context, the clinical plausibility for carcinogenesis is the chronic phase, often after repeated malaria infection, especially when Hz-laden macrophages and dendritic cells persist despite parasite clearance. The connection between chronic inflammation and cancer has been widely reported in different malignancies such as colon, liver, stomach and prostate cancers (29, 30). A microenvironment of inflammation promotes carcinogenesis by maintaining the generation of cytokines, chemokines, reactive species, and growth factors that affect cell survival, cell proliferation, and mutation rates (8, 31).

Chronic inflammation, in contrast to acute inflammation, is one that has been activated over a long period and may not be resolved (32, 33). The persistent presence of hemozoin (Hz) in the immune cells, like macrophages and dendritic cells, following the elimination of malaria parasites, is a major and sustained inflammatory stimulus (34, 35). It has been found that Hz can stimulate such signalling pathways as NF-kB (nuclear factor kappa-light-chain-enhancer of activated B cells) and MAPKs (mitogen-activated protein kinases), which subsequently increase the expression of genes that mediate cytokine production (e.g., TNF-α, IL-1β, IL-6), cell survival, and angiogenesis (36, 37).

**Figure 3:**
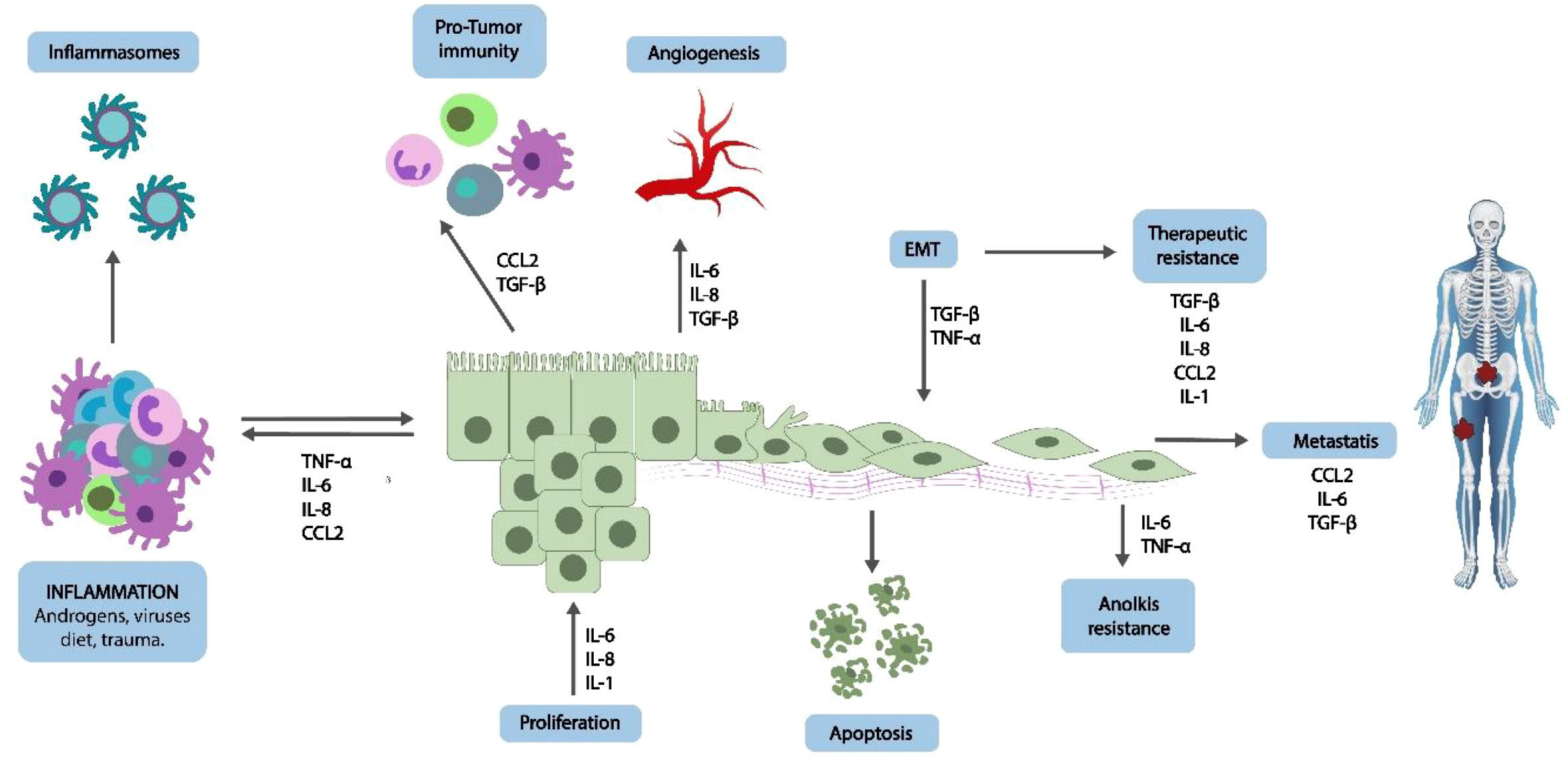
Hemozoin-induced chronic inflammation and prostate carcinogenesis. Adapted from (38).

These proinflammatory cytokines are also important in attracting immune cells as well as influencing the epithelial cells in the local microenvironment, as depicted in **Figure 3**. Prolonged exposure of the prostate to inflammatory cytokines may result in hyperplasia of epithelial cells, which predispose the appearance of DNA damage and epigenetic alterations that could cause cancer (39). Among the most important consequences of chronic inflammation is the excessive production of reactive oxygen species (ROS) and reactive nitrogen species (RNS). Hemozoin-laden macrophages can exhibit sustained low-grade oxidative burst and cytokines production after secondary stimulation leads to chronic inflammatory signalling in distant tissues (40, 41).

These reactive molecules are also necessary to combat the pathogens; however, they may also have an unintended consequence of damaging host cells through oxidative damage to DNA, proteins, and lipids (28, 42). Otherwise, the DNA damage, including 8-oxoguanine or single-strand breaks, cross-linking could result in mutations (43). The prolonged exposure to NO may result in the nitrosative deamination of DNA bases and disrupt the mechanism of functioning of DNA repair enzymes. This further enhances the genomic instability (40).

Chronic inflammation is a key player in shaping the tumour microenvironment (TME). Inflammatory cytokines set off angiogenesis by triggering vascular endothelial growth factor (VEGF), which helps tumours grow and spread (41, 44). Moreover, continuous exposure to hemozoin (Hz) can lead macrophages to take on the M2 phenotype, which weakens cytotoxic immune responses and promotes tissue remodelling and tumour progression (39, 41). This immunosuppressive setting allows precancerous cells to slip past the immune system, a crucial aspect of tumour development (28). The prostate is an area notorious for its low immunosurveillance and high susceptibility to inflammation-related damage; this mechanism could significantly heighten cancer risk (45). The inflammation caused by hemozoin shares similar pathways with other well-known inflammation-cancer connections, like Helicobacter pylori in gastric cancer, Hepatitis B/C viruses in liver cancer, and Schistosoma haematobium in bladder cancer. In all these cases, chronic exposure to inflammatory mediators leads to changes in epithelial cells (46, 47), as summarised in **Table 3**.

**Table 3:** Inflammation-driven mechanisms of carcinogenesis across pathogens.

Pathogen/toxin	Associated cancer	Inflammatory mediators	Mechanisms involved
*Plasmodium* (Hemozoin)	Prostate (proposed)	TNF-α, IL-6, NO, ROS	DNA damage, NF-κB activation, M2 macrophages
*Helicobacter pylori*	Gastric carcinoma	IL-8, TNF-α, ROS	DNA damage, epithelial cell proliferation
HBV/HCV	Hepatocellular carcinoma	IL-6, TNF-α, ROS, TGF-β	Chronic hepatitis, fibrosis, immune suppression
*Schistosoma haematobium*	Bladder cancer	IL-6, eosinophilic inflammation	Granuloma formation, urothelial hyperplasia
Asbestos/silica	Lung cancer	IL-1β, TNF-α, ROS	Fibrosis, chronic macrophage activation

The inflammatory response triggered by hemozoin has a lot in common with the well-known processes that lead to cancer due to inflammation. By maintaining cytokine signalling, causing oxidative stress, and modulating the immune system, hemozoin could significantly transform prostate epithelial cells and create an environment that promotes tumour growth. Although the direct links between malaria-related hemozoin and prostate cancer are still being studied, the biological evidence suggests that this is a topic worth investigating further.

## 5 Immunomodulatory effects of hemozoin in the prostate microenvironment

Although the *Plasmodium* parasites are not directly attacking the prostate per se, the outcome of the exposure to hemozoin (Hz) may have an indirect effect on the immune environment in the prostate as a result of continuous inflammation and immune system alterations. The immune-privileged condition of the prostate and its sensitivity to cytokine-mediated inflammation are widely known; even small modifications in the peripheral immune system can dramatically alter the local tumour microenvironment (TME), which can result in the formation of cancer (48, 49). Persistent malaria infections, especially in endemic regions like Asia and SSA, can potentially produce a cumulative Hz circulation in tissue resident phagocytes. Over time, these cells interact with the microenvironment of the prostate by exporting chronic inflammatory signals initiated by Organs accumulating Hz (spleen, liver, bone marrow) to the prostate.

The hemozoin remains much longer than the parasites, and it is found in monocytes, macrophages, and dendritic cells (DCs) and affects cell behaviour. Studies have shown that long-term exposure to Hz may divert the immune response towards an immunosuppressive and tumour-favouring pattern (8, 28). This immunological change can trigger a cascade in the prostate, which is particularly susceptible to cytokines of inflammatory origin and hormonal stimuli (25). Although Hz exhibits a direct effect on cytokine secretion *in vitro* assays, persistent accumulation of Hz within the monocytes and dendritic cells can reprogram cells towards an immunosuppressive tumour-promoting phenotype over time. This impact is more captured in its long term effects on immune cell differentiation and response.

**Figure 4:**
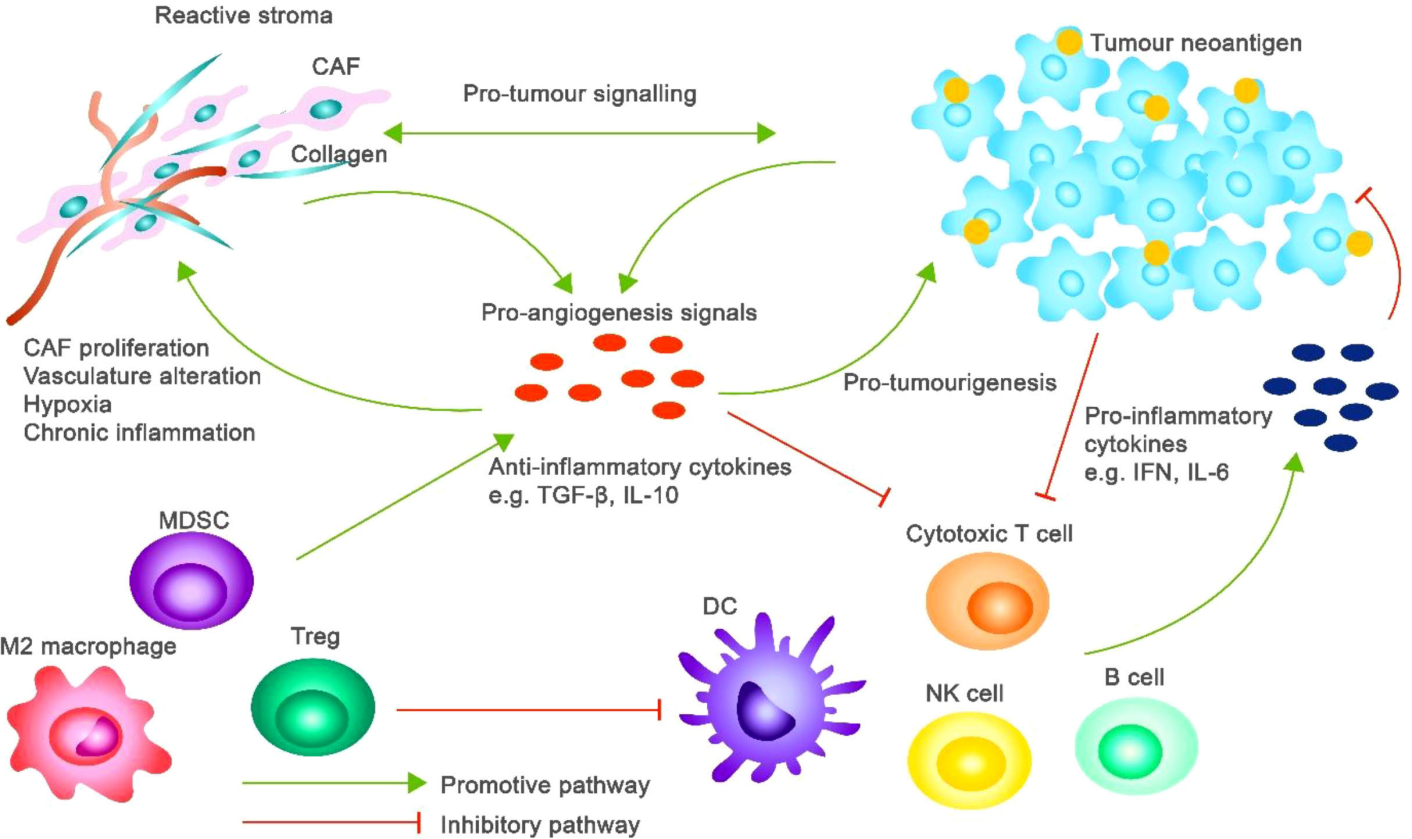
Hemozoin-induced immune reprogramming in the prostate microenvironment. A conceptual diagram that shows how Hz can lead to macrophage polarisation towards the M2 type, which boosts the production of IL-10 and TGF-β. This process suppresses CD8+ T cells and promotes the expansion of Tregs, ultimately facilitating immune evasion and aiding in the progression of prostate tumours. Adapted from (42).

However, a significant immune change associated with Hz is the transition of macrophages from the M1 (pro-inflammatory) to the M2 (immunosuppressive) type. M2 macrophages produce anti-inflammatory cytokines such as IL-10 and TGF-β, diminish antigen presentation, and encourage tissue remodelling and blood vessel formation, all of which can support tumour growth (39, 50), as illustrated in **Table 4**; **Figure 4**. In the prostate, the presence of M2-like tumour-associated macrophages (TAMs) is associated with worse outcomes and more aggressive tumour behaviour (41, 45).

**Table 4:** Functional differences between M1 and M2 macrophages in the context of prostate cancer.

Feature	M1 macrophages	M2 macrophages
Cytokines produced	IL-12, TNF-α, IL-6	IL-10, TGF-β, CCL17
Role in cancer	Anti-tumour (immune activation)	Pro-tumour (immunosuppression)
Antigen presentation	High	Low
Angiogenesis	Inhibits	Promotes
Presence in prostate tumours	Rare	Common, especially in late-stage PCa

Hemozoin (Hz) is a crystalline byproduct formed when Plasmodium digests haemoglobin. It can significantly disrupt T-cell functions, particularly the CD8^+^ cytotoxic T lymphocytes (CTLs) that play a crucial role in identifying and destroying newly formed cancer cells. When the immune system encounters Hz, it tends to become more suppressive, mainly due to the effects of interleukin-10 (IL-10) and transforming growth factor-beta (TGF-β). This interaction results in a decrease in CTL proliferation, interferon-gamma (IFN-γ) production, and overall cytolytic activity. As a result, immune surveillance in areas like the prostate takes a significant hit (35, 51). Moreover, Hz has been found to hinder the maturation of dendritic cells and their ability to present antigens, which further weakens T-cell priming and activation, allowing transformed epithelial cells to evade the immune system (35).

Hemozoin might also play a role in increasing the number of Tregs (CD4^+^CD25^+^FoxP3^+^ cells), which suppress the activity of effector T cells and help maintain tolerance in the periphery **Figure 4**. In the tumour microenvironment, higher levels of Tregs are a clear sign of immune evasion and are often linked to poorer survival rates in prostate cancer patients (52). The cytokine TGF-β, which is produced by macrophages loaded with Hz, is crucial for the differentiation and expansion of Tregs (53).

The IL-10 and TGF-β are the cytokines triggered by hemozoin (Hz) and are critical in facilitating epithelial-mesenchymal transition (EMT) in prostate epithelial cells. This change allows the epithelial cells to gain migratory and invasive properties, and this is a critical process leading to metastasis (45). Besides, M2 macrophages and Tregs play a role in angiogenesis through the release of pro-angiogenic cytokines such as VEGF, FGF, and matrix metalloproteinases (MMPs), which favour tumour growth and blood vessel growth (45, 54). The immunomodulatory action of hemozoin can be observed beyond the immediate immune reaction to create a long-term immunosuppressive condition, which can make remote tissues, like the prostate, susceptible to neoplastic alterations. Hz impairs immune balance by reprogramming macrophages, suppressing CTLs, and increasing the number of Tregs, which provides favourable conditions leading to tumour progression, EMT, and angiogenesis. These changes, though indirectly associated with a by-product of malaria, may turn out to be a different cause of prostate cancer in such areas where malaria is common (9).

## 6 Molecular signalling pathways

Hemozoin (Hz) is a pigment that is relatively old and is associated with malaria as well as a by-product of haemoglobin metabolism. But it is providing interest as an effective immunomodulatory agent that has the ability to interfere with a number of molecular signalling pathways (35, 55). Whenever Hz lingers in host immune cells, especially in the macrophages and dendritic cells, it can initiate a cascade of intracellular pathways, which overlap with those commonly related to the formation of prostate cancer (40, 41). The most important pathways taken into account are: TLR/MyD88/NF-kB, PI3K/Akt/mTOR, and STAT3, which could be improperly stimulated by the exposure to Hz that could cause detrimental alterations in the microenvironment of the prostate (8, 40).

**Figure 5:**
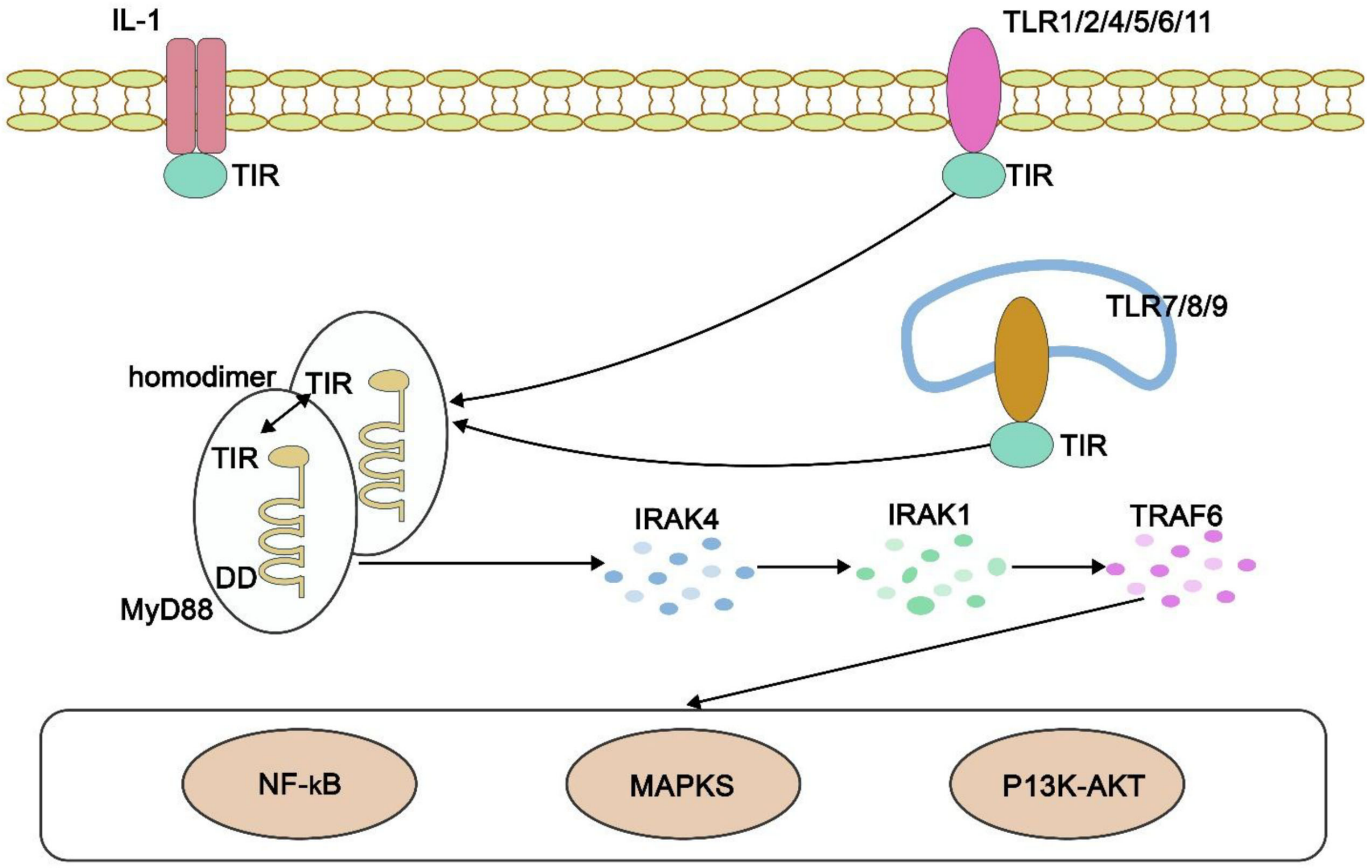
Hz phagocytosis leading to TLR9 activation, downstream signalling through MyD88, and subsequent activation of NF-κB, PI3K/Akt/mTOR, and STAT3 pathways. Adapted from (56).

The Toll-like receptor (TLR) family is also critical in the innate immune response as it identifies pathogen-associated molecular patterns (PAMP) and danger-associated molecular patterns (DAMP). Hemozoin itself is a DAMP, which is known to activate TLR9 in macrophage and dendritic cells **Figure 5**, which activates MyD88-dependent signalling (35, 57). The mediation of this interaction results in phosphorylation and translocation of NF-kB into the nucleus, which is a transcription factor that promotes the expression of pro-inflammatory cytokines such as TNF-α, IL-1β, and IL-6 (9, 37, 55). Furthermore, Hz induction of TLR has also been linked with the release of reactive oxygen species (ROS) and NLRP3 inflammasome, which are both connected to chronic inflammation (8, 40).

In the context of prostate cancer:

- Chronic NF-κB activation has been associated with increased tumour cell survival, angiogenesis, and resistance to apoptosis (9, 58).
- NF-κB also facilitates the recruitment and polarisation of M2 macrophages and regulatory T cells, compounding the immunosuppressive microenvironment established by Hz (39–41).

This environment not only helps the immune system evade detection but also encourages a process called epithelial-mesenchymal transition (EMT) and promotes metastatic behaviour in prostate epithelial cells (59).

The PI3K/Akt/mTOR pathway is frequently disrupted in prostate cancer, often because of the loss of PTEN or the amplification of Akt. Additionally, inflammatory mediators triggered by Hz, such as IL-6 and TNF-α, can further enhance this pathway:

- IL-6 activates PI3K, leading to Akt phosphorylation, which promotes cell growth, metabolism, and survival.
- Activated Akt then stimulates mTOR, enhancing protein synthesis and angiogenesis via HIF-1α and VEGF upregulation.

It’s important to note that when the PI3K/Akt/mTOR pathway is constantly activated, it not only promotes cell growth but also boosts resistance to anti-androgen treatments (60). The inflammatory environment created by hemozoin could speed up the progression of prostate tumours that are driven by hormones. The Signal Transducer and Activator of Transcription 3 (STAT3) is a transcription factor that gets activated by a range of cytokines, especially IL-6, which is released in large amounts during immune activation triggered by hemozoin. Upon activation, STAT3 translocates to the nucleus, where it regulates genes involved in:

- Cell proliferation (e.g., c-Myc, Cyclin D1).
- Immune suppression (e.g., IL-10, PD-L1).
- Invasion and metastasis (e.g., MMPs, VEGF).

Persistent STAT3 signalling has been associated with a poor prognosis, immune evasion, and resistance to treatment in prostate cancer (61). In light of the cytokine-rich environment created by Hz, STAT3 activation could be a crucial factor linking malaria-related inflammation to the development of prostate cancer, as summarised in **Table 5**.

**Table 5:** Summary of key molecular pathways activated by hemozoin and their oncogenic roles in prostate cancer.

Pathway	Activation triggered by Hz	Key oncogenic effects in prostate cancer	Principal mediators
TLR/MyD88/NF-κB	TLR9 activation by Hz	Cytokine release, apoptosis inhibition, and immune evasion	TNF-α, IL-6, Bcl-xL
PI3K/Akt/mTOR	IL-6, TNF-α, oxidative stress	Cell growth, survival, and therapy resistance	Akt, mTOR, HIF-1α, VEGF
STAT3	IL-6, IL-10	Proliferation, immune suppression, angiogenesis	Cyclin D1, PD-L1, VEGF

These signalling cascades are not isolated but interconnected, with significant crosstalk:

- NF-κB can enhance STAT3 transcriptional activity, reinforcing inflammation and immune evasion.
- PI3K/Akt signalling can stabilise NF-κB and promote STAT3 activation, forming a feedback loop that supports tumorigenesis.

Understanding how Hz integrates into this network opens up potential therapeutic strategies:

- TLR antagonists may prevent aberrant NF-κB activation.
- JAK/STAT inhibitors could be used to block chronic STAT3 signalling.
- mTOR inhibitors might counteract Hz-fuelled Akt activation.

Hemozoin plays a role in prostate cancer development by continuously activating certain molecular signalling pathways that are known to aid in tumour growth, immune suppression, and resistance to treatment (62). By affecting the TLR/MyD88/NF-κB, PI3K/Akt/mTOR, and STAT3 pathways, hemozoin sets the stage for cancer development and progression. By concentrating on these pathways in regions where malaria is prevalent, we could uncover new strategies for preventing or treating cancer in vulnerable populations.

## 7 Epidemiological perspectives and geographic correlation

Malaria has been a major public health challenge in most tropical and subtropical regions, particularly in Sub-Saharan Africa, Southeast Asia, and some regions of South America **Table 6**. Interestingly, cases and deaths of prostate cancer have also increased tremendously in the regions in the recent decades, which gives rise to the question of whether malaria exposure has any relation with the occurrence of prostate cancer (63). Numerous epidemiological research studies have revealed that there is a positive relationship between the prevalence of malaria and the rising prevalence of prostate cancer at the population level. Indicatively, nations with a high rate of malaria caused by Plasmodium falciparum have been reported to record a higher prostate cancer rate than other areas with little or no malaria transmission (63). Nevertheless, it is rather complex to understand how the immunomodulation under the influence of hemozoin is put into this context and what other factors also play roles in this process. Factors such as health care facilities, screening habits, lifestyle and genetics can contribute substantially to the cancer rates.

Besides, longitudinal cohort surveys and case-control studies indicate that recurrent malaria infections resulting in chronic retention of hemozoin in immune cells may be a factor in the systemic impairment of immune regulation. It is thought that this chronic inflammatory condition may provide an environment that may damage prostate epithelial DNA and promote the development of oncogenic alterations. Therefore, the chronic malaria-induced long-term immunological impacts can serve as a confounding variable to develop prostate cancer, particularly in areas where the disease is very widespread (63). This correlation is indeed evident in a side-by-side comparison of malaria-endemic and malaria-non-endemic areas. **Table 6** presents the incidence and mortality of prostate cancer and the prevalence of malaria in different countries. The most notable is that regions that have a greater hemozoin burden, as we can deduce by the intensity of malaria transmission, tend to have an increased risk of prostate cancer. It is important to note.

**Table 6:** Comparative epidemiological data of malaria burden and prostate cancer incidence.

Country/region	Malaria prevalence (%)	Estimated hemozoin burden	Prostate cancer incidence (per 100,000)	Prostate cancer mortality (per 100,000)
Nigeria	25	High	38.1	18.7
Kenya	15	Moderate	25.4	11.3
South Africa	2	Low	33.5	12.0
United States	<0.01	Negligible	97.2	18.5
United Kingdom	<0.01	Negligible	56.3	10.4

*Source* (63, 64).

The incidence of malaria has declined over the years in many endemic countries, whereas prostate cancer incidence has increased over the same period. The incidence has been driven mainly by an ageing population, increased access to PSA testing and awareness. Thus, the contribution of Hz-mediated immunomodulation is likely superimposed on demographic and health system changes rather than a parallel relationship due to increased malaria and cancer incidence. However, additional comprehensive epidemiological research that considers socioeconomic, genetic, and environmental aspects is needed. It would be useful to conduct high-population-based research that would integrate malaria surveillance data with cancer registries (63).

As highlighted in **Table 6** above, malaria-endemic countries like Nigeria also have substantial prostate cancer incidence and mortality; in addition, non-endemic countries with little to no malaria incidence, like the USA and UK, exhibit even higher prostate cancer incidence. These patterns highlight that Hz exposure is not necessarily a prominent prostate cancer disease-causing factor at a population level. However, malaria-endemic regions with recurrent malaria-induced inflammations and immune alterations may be a cofactor alongside a plethora of cofactors that put individuals and populations at risk. In addition, several coinfections also interact with Hz-laden immunity, which may make it difficult to disentangle the contribution of Hz from other contributing inflammatory response drivers. Furthermore, though there exist epidemiological findings that agree with the potential contributory role of malaria-associated Hz in prostate cancer carcinogenesis, the evidence is not substantial enough. While there’s some solid epidemiological evidence pointing to a link between malaria prevalence and a rise in prostate cancer cases, we really need more thorough studies across various fields to pin down those causal relationships. Thus, carefully designed case-controlled and longitudinal cohort studies are required to test this hypothesis. These studies should assess malaria history, biomarkers of Hz exposure, and other confounders.

## 8 Therapeutic and diagnostic implications

The study of the role of hemozoin (Hz) in the development of the immune system and its potential relationship with prostate cancer presents some exciting possibilities for novel treatment and diagnostic methodology. As Hz can modify immune signalling and create a pro-tumour environment, a better understanding of the pathways modulated by Hz would, indeed, make our response to prostate cancer much more effective, particularly in areas where chronic malaria is the norm. A highly interesting approach involves the modulation of the Toll-like receptor (TLR) pathways, especially that of TLR9, which is stimulated by Hz. We can perhaps reduce the chronic inflammation and immune imbalances that lead to prostate tumour development by pharmacological inhibition or pharmacological modulation of TLR9 and its downstream signalling partners, such as MyD88 and NF-kB. Moreover, restoration of effective immune surveillance and reduced tumour growth by re-polarising macrophages, which are pro-tumour M2, into tumour-fighting M1, may also be included.

More targets also can be found in the PI3K/Akt/mTOR and STAT3 pathways that are commonly impaired in prostate cancer and worsened by the inflammation of Hz. Small-molecule inhibitors and monoclonal antibodies targeting these pathways are undergoing research or are already used in the treatment of different cancers and might be adapted or improved to be used in malaria-endemic regions (65, 66). A combination of anti-inflammatory agents with the usual treatment of prostate cancer, including the androgen deprivation therapy or chemotherapy, may be even more effective by improving the situation by dealing with both tumour development and immune dysfunction linked with Hz. Diagnostically, the occurrence of biomarkers associated with Hz exposure, as well as its effects on the immune system, may result in a more effective early diagnosis and risk evaluation. As an example, the analysis of Hz-induced cytokines, such as the increase in IL-10, TGF-β, TNF-α, and IL-6, in plasma or tissues, may display a tumour-friendly environment (36, 67). The proposed biomarkers can be examined to ascertain if Hz-related signatures add independent predictive value to the correlation of Hz to prostate carcinogenesis. Additionally, immune phenotypes with an upsurge of regulatory T cells (Tregs) or polarisation of macrophages might be added to liquid biopsy assays or tissue-based assays. Such biomarkers would be highly useful in regions with malaria scourges, where funds could be limited to prostate cancer screening. Diagnostic model A comprehensive diagnostic model that would combine history of infectious diseases (frequency and severity of malaria episodes), levels of hemozoin (which could be detected using certain specific imaging or biochemical tests), and the presence of inflammatory changes and immune response (measured with the help of some specific markers) would help a great deal to complement the risk prediction models of prostate cancer. Such a methodology would enable the personalised intervention and preventive measures to be used with people at increased risk.

## 9 Future directions

The research should be aimed at the development of non-invasive tests that will help identify hemozoin or its immunological markers, such as circulating extracellular vesicles carrying Hz components or microRNAs induced by Hz. In addition, long-term research findings of cases of malaria survivors followed up to determine the frequency of cellular changes induced by prostate cancer and how their immunological statuses are altered will be important in confirming these diagnostic tools. Conclusively, integrating the information we have on the role of hemozoin on the immune system into prostate cancer treatment would be a step to more effective treatment and new ways of diagnosis, particularly for people who live in malaria-afflicted regions. These possibilities will require a cooperative strategy that unites specialists in the fields of oncology, immunology, and infectious diseases.
